# Supplementary material for: Perioperative Probiotics Application for Preventing Postoperative Complications in Patients with Colorectal Cancer: A Systematic Review and Meta-Analysis
Source: Medicina (Kaunas). 2022 Nov 14;58(11):1644. doi: 10.3390/medicina58111644 (PMC9699544; doi:10.3390/medicina58111644)
Supplement: Supplementary file 1 [file medicina-58-01644-s001.zip › supplementary_file_3_search_strategy.pdf]

Table S3. Search strategy.

Medline via Ovid
1. exp Colorectal Neoplasms/ or exp Colonic Neoplasms/ or exp Rectal Neoplasms/ or exp Sigmoid Neoplasms/<br>2. ((cancer* or carcinoma* or neoplasm* or adenoma* or adenocarcinom* or tumour* or tumor* or malignan*) adj3 (colorectal* or colon* or rect* or sigmoid colon*)).mp.<br>3. 1 or 2<br>4. exp Probiotics/<br>5. exp Synbiotics/<br>6. exp Lactobacillus/<br>7. exp Bifidobacterium/<br>8. exp Lactococcus/<br>9. exp Saccharomyces/<br>10. exp Enterococcus/<br>11. exp Yeast, dried/<br>12. exp Pediococcus/<br>13. exp Leuconostoc/<br>14. exp Bacillus subtilis/<br>15. exp Yogurt/

16. exp Cultured milk products/
17. exp Streptococcus thermophilus/
18. exp Antibiosis/
19. (probiotic* or synbiotic* or Lactobacillus or Bifidobacterium or Lactococcus or Saccharomyce* or Enterococcus or (dried adj1 yeast) or Pediococcus or Leuconostoc or Bacillus subtilis or Yogurt or ((Cultured or fermented) adj2 milk) or Streptococcus thermophilus or microbial dietary supplement* or Bulgarian bacillus or microbial antagonism* or bacterial interference*).mp.
20. 4 or 5 or 6 or 7 or 8 or 9 or 10 or 11 or 12 or 13 or 14 or 15 or 16 or 17 or 18 or 19
21. 3 and 20
22. randomized controlled trial.pt.
23. controlled clinical trial.pt.
24. randomized.ab.
25. placebo.ab.
26. drug therapy.fs.
27. randomly.ab.
28. trial.ab.
29. groups.ab.
30. 22 or 23 or 24 or 25 or 26 or 27 or 28 or 29
31. exp animals/ not humans.sh.

32. 30 not 31<br><br>33. 21 and 32
Embase (via Elsevier)
1. 'colorectal tumor'/exp<br>2. ((colorectal* OR colon* OR rect* OR 'sigmoid colon*') NEAR/3 (cancer* OR carcinom* OR tumor* OR tumour* OR neoplasm* OR adeno* OR neoplasia* OR malignan*)):ti,ab<br>3. #1 OR #2<br>4. 'probiotic agent'/exp OR 'synbiotic agent'/exp OR 'lactobacillus'/exp OR 'bifidobacterium'/exp OR 'lactococcus'/exp OR 'saccharomyces'/exp OR 'enterococcus'/exp OR 'dried yeast'/exp OR 'pediococcus'/exp OR 'leuconostoc'/exp OR 'bacillus subtilis'/exp OR 'yoghurt'/exp OR 'fermented dairy product'/exp OR 'streptococcus thermophilus'/exp OR 'antibiosis'/exp<br>5. probiotic*:ti,ab OR synbiotic*:ti,ab OR lactobacillus:ti,ab OR bifidobacterium:ti,ab OR lactococcus:ti,ab OR saccharomyce*:ti,ab OR enterococcus:ti,ab OR ((dried NEAR/1 yeast):ti,ab) OR pediococcus:ti,ab OR leuconostoc:ti,ab OR 'bacillus subtilis':ti,ab OR yogurt:ti,ab OR (((cultured OR fermented) NEAR/2 milk):ti,ab) OR 'streptococcus thermophilus':ti,ab OR 'microbial dietary supplement*':ti,ab OR 'bulgarian bacillus':ti,ab OR 'microbial antagonism*':ti,ab OR 'bacterial interference*':ti,ab<br>6. #4 OR #5

7. #3 AND #6<br>8. 'crossover procedure':de OR 'double-blind procedure':de OR 'randomized controlled trial':de OR 'single-blind procedure':de OR random*:de,ab,ti OR factorial*:de,ab,ti OR crossover*:de,ab,ti OR ((cross NEXT/1 over*):de,ab,ti) OR placebo*:de,ab,ti OR ((doubl* NEAR/1 blind*):de,ab,ti) OR ((singl* NEAR/1 blind*):de,ab,ti) OR assign*:de,ab,ti OR allocat*:de,ab,ti OR volunteer*:de,ab,ti<br>9. 'animals'/exp NOT ('humans'/exp AND 'animals'/exp)<br>10. #8 NOT #9<br>11. #7 AND #10
Cochrane via Wiley
1. [mh "Colorectal Neoplasms"] or [mh "Colonic Neoplasms"] or [mh "Rectal Neoplasms"] or [mh "Sigmoid Neoplasms"]<br>2. ((colorectal* or colon* or rect* or "sigmoid colon*") near/3 (cancer* or carcinom* or tumor* or tumour* or neoplasm* or adeno* or neoplasia* or malignan*)):ti,ab,kw<br>3. #1 or #2<br>4. [mh "Probiotics"] or [mh "Synbiotics"] or [mh "Lactobacillus"] or [mh "Bifidobacterium"] or [mh "Lactococcus"] or [mh "Saccharomyces"] or [mh "Enterococcus"] or [mh "Yeast, dried"] or [mh "Pediococcus"] or [mh "Leuconostoc"] or [mh "Bacillus subtilis"] or [mh "Yogurt"] or [mh "Cultured

milk products"] or [mh "Streptococcus thermophilus"] or [mh "Antibiosis"]<br>5. (probiotic* or synbiotic* or lactobacillus or bifidobacterium or lactococcus or saccharomyce* or enterococcus or ((dried NEAR/1 yeast)) or pediococcus or leuconostoc or "bacillus subtilis" or yogurt or (((cultured or fermented) near/2 milk)) or "streptococcus thermophilus" or "microbial dietary supplement*" or "bulgarian bacillus" or "microbial antagonism*" or "bacterial interference*"):ti,ab,kw<br>6. #4 or #5<br>7. #3 and #6 #6 and #10
Scopus
TITLE-ABS-KEY((colorectal* OR colon* OR rect* OR "sigmoid colon*") W/3 (cancer* OR carcinom* OR tumor* OR tumour* OR neoplasm* OR adeno* OR neoplasia* OR malignan*)) AND TITLE-ABS-KEY(probiotic* OR synbiotic* OR lactobacillus OR bifidobacterium OR lactococcus OR saccharomyce* OR enterococcus OR "dried yeast" OR pediococcus OR leuconostoc OR "bacillus subtilis" OR yogurt OR ((cultured OR fermented) W/2 milk) OR "streptococcus thermophilus" OR "microbial dietary supplement*" OR "bulgarian bacillus" OR "microbial antagonism*" OR "bacterial interference*") AND (INDEXTERMS ("clinical trials" OR "clinical trials as a topic" OR "randomized controlled trial" OR "Randomized Controlled Trials as Topic" OR "controlled clinical trial"

OR "Controlled Clinical Trials" OR "random allocation" OR "Double-Blind Method" OR "Single-Blind Method" OR "Cross-Over Studies" OR "Placebos" OR "multicenter study" OR "double blind procedure" OR "single blind procedure" OR "crossover procedure" OR "clinical trial" OR "controlled study" OR "randomization" OR "placebo")) OR (TITLE-ABS-KEY (("clinical trials" OR "clinical trials as a topic" OR "randomized controlled trial" OR "Randomized Controlled Trials as Topic" OR "controlled clinical trial" OR "Controlled Clinical Trials as Topic" OR "random allocation" OR "randomly allocated" OR "allocated randomly" OR "Double-Blind Method" OR "Single-Blind Method" OR "Cross-Over Studies" OR "Placebos" OR "cross-over trial" OR "single blind" OR "double blind" OR "factorial design" OR "factorial trial"))) OR (TITLE-ABS (clinical AND trial* OR trial* OR rct* OR random* OR blind*)) AND NOT INDEX (medline)
Web of Science
1. TS=((colorectal* OR colon* OR rect* OR "sigmoid colon*") NEAR/3 (cancer* OR carcinom* OR tumor* OR tumour* OR neoplasm* OR adeno* OR neoplasia* OR malignan*))<br>2. TS=(probiotic* OR synbiotic* OR lactobacillus OR bifidobacterium OR lactococcus OR saccharomyce* OR enterococcus OR "dried yeast" OR

pediococcus OR leuconostoc OR "bacillus subtilis" OR yogurt OR ((cultured OR fermented) NEAR/2 milk) OR "streptococcus thermophilus" OR "microbial dietary supplement*" OR "bulgarian bacillus" OR "microbial antagonism*" OR "bacterial interference*")<br>3. TS=(randomised OR randomized OR randomisation OR randomisation OR placebo* OR (random* AND (allocat* OR assign*)) OR (blind* AND (single OR double OR treble OR triple)))<br>4. #1 AND #2 AND #3
LILACS
1. (colorectal* OR colon* OR rect* OR "sigmoid colon*") AND (cancer* OR carcinoma* OR neoplasm* OR adenoma* OR adenocarcinom* OR tumour* OR tumor* OR malignan*) AND (probiotic* OR synbiotic* OR lactobacillus OR bifidobacterium OR lactococcus OR saccharomyce* OR enterococcus OR "dried yeast" OR pediococcus OR leuconostoc OR "bacillus subtilis" OR yogurt OR "cultured milk" OR "fermented milk" OR "streptococcus thermophilus" OR "microbial dietary supplement*" OR "bulgarian bacillus" OR "microbial antagonism*" OR "bacterial interference*")
ClinicalTrials.gov
1. (Colorectal OR colon) AND Cancer<br>2. probiotic* OR synbiotic* OR lactobacillus OR bifidobacterium OR lactococcus OR saccharomyce* OR enterococcus OR "dried yeast" OR

pediococcus OR leuconostoc OR "bacillus subtilis" OR yogurt<br>3. 1 AND 2
World Health Organization International Clinical Trials Registry Platform search portal
1. (Colorectal OR colon) AND probiotic*
Grey Literature (Open Grey)
1. (Colorectal OR colon) AND probiotic*
