# Supplementary material for: Perioperative Probiotics Application for Preventing Postoperative Complications in Patients with Colorectal Cancer: A Systematic Review and Meta-Analysis
Source: Medicina (Kaunas). 2022 Nov 14;58(11):1644. doi: 10.3390/medicina58111644 (PMC9699544; doi:10.3390/medicina58111644)
Supplement: Supplementary file 1 [file medicina-58-01644-s001.zip › supplementary_file_4_cha_inc_studies.pdf]

Table S4. Characteristics of included studies (ordered by study ID).

A. Study ID for included studies

Study ID	Identified articles
Park 2020 [24]	* Park IJ, Lee JH, Kye BH, Oh HK, Cho YB, Kim YT, et al. 2020 Lee; Effects of PrObiotics on the Symptoms and Surgical ouTComes after Anterior REsection of Colon Cancer (POSTCARE): A Randomized, Double-Blind, Placebo-Controlled Trial. J Clin Med. 2020;9(7).<br>• Lee I.K, Kye B.H, Lee J.H, Park I.J, Oh H.K, Cho Y.B. et al. THE EFFECTS OF PROBIOTICS ON SYMPTOM AND SURGICAL OUTCOME AFTER ANTERIOR RESECTION OF COLON CANCER; MULTICENTER, DOUBLE-BLIND, RANDOMIZED, PLACEBO-CONTROLLED TRIAL (POSTCARE STUDY). Gastroenterology 2019 156:6 (S-1423) Supplement 1<br>• The Effects of Mechnikov Probiotics on Symptom and Surgical Outcome. https://clinicaltrials.gov/show/NCT03531606
Polakowskin 2019 [14]	* Polakowski CB, Kato M, Preti VB, Schieferdecker MEM, Ligocki Campos AC. 2019 Polakowski; Impact of the preoperative use of synbiotics in colorectal cancer patients: A prospective, randomized, double-blind, placebo-controlled study. Nutrition. 2019;58:40-6.
Bajramagic 2019 [25]	* Bajramagic S, Hodzic E, Mulabdic A, Holjan S, Smajlovic SV, Rovcanin A. 2019 Bajramagic; Usage of Probiotics and its Clinical Significance at Surgically Treated Patients Sufferig from Colorectal Carcinoma. Med Arch. 2019;73(5):316-20.
Flesche 2017 [26]	* Flesch AT, Tonial ST, Contu PC, Damin DC. 2017 Flesche; Perioperative synbiotics administration decreases postoperative infections in patients with colorectal cancer: a randomized, double-blind clinical trial. Rev Col Bras Cir. 2017;44(6):567-73.
Yang 2016 [15]	* Yongzhi Yang, Yang Xia, and Yanlei Ma, Hongqi Chen, Leiming Hong, Junlan Feng, Yang ZY, Chenzhang Shi1, Wen Wu1, Renyuan Gao, Qing Wei Qin. The effect of perioperative probiotics treatment for colorectal cancer: short-term outcomes of a randomized controlled trial. Oncotarget. 2016;7(7).<br>• Effects of perioperative probiotics treatment on fecal microflora, gut barrier function and complications in colorectal cancer surgery: a prospective randomized clinical trial http://www.chictr.org.cn/showproj.aspx?proj=6227
Theodoropoulos 2016 [27]	* Theodoropoulos GE, Memos NA, Peitsidou K, Karantanos T, Spyropoulos BG, Zografos G. Synbiotics and gastrointestinal function-related quality of life after elective colorectal cancer resection. Ann Gastroenterol. 2016;29(1):56-62.<br>• Theodoropoulos, G.; Memos, N.; Peitsidou, K.; Marinatou, A.; Zografos, G. Effects of synbiotics on gastrointestinal function after colectomy for cancer: results from a prospective randomized trial (NCT01479907)<br>• Synbiotics and Gastrointestinal Function Related Quality of Life After Colectomy for Cancer. https://clinicaltrials.gov/ct2/show/NCT01479907
Tan 2016 [16]	* Tan CK, Said S, Rajandram R, Wang Z, Roslani AC, Chin KF. Pre-surgical Administration of Microbial Cell Preparation in Colorectal Cancer Patients: A Randomized Controlled Trial. World J Surg. 2016;40(8):1985-92.<br>• Does the Pre-operative Use of Probiotic Prevent Ileus In Patients with Colorectal Cancer After Surgery? A Randomized, Double- Blind, Placebo-Controlled Trial.

	https://anzctr.org.au/Trial/Registration/TrialReview.aspx?ACTRN=12615000545561
Mizuta 2016 [28]	* Mizuta. 2016 Mizuta; Perioperative supplementation with bifidobacteria improves postoperative nutritional recovery inflammatory response, and faecal microbiota in patients undergoing colorectal surgery: a prospective, randomized clinical trial (2016). 2016.
Krebs 2016 [29]	* Krebs B. Prebiotic and Synbiotic Treatment before Colorectal Surgery--Randomised Double Blind Trial. Coll Antropol. 2016;40(1):35-40.<br>• Krebs B; Horvat M; Golle A; Krznaric Z; Papes D; Augustin G, et al. A randomized clinical trial of synbiotic treatment before colorectal cancer surgery. American Surgeon. 79(12):E340-2, 2013 Dec.
Komatsu 2016 [17]	* Komatsu S, Sakamoto E, Norimizu S, Shingu Y, Asahara T, Nomoto K, et al. Efficacy of perioperative synbiotics treatment for the prevention of surgical site infection after laparoscopic colorectal surgery: a randomized controlled trial. Surg Today. 2016;46(4):479-90.
Kotzampassi 2016 [18]	* Kotzampassi K, Stavrou G, Damoraki G, Georgitsi M, Basdanis G, Tsaousi G, et al. A Four-Probiotics Regimen Reduces Postoperative Complications After Colorectal Surgery: A Randomized, Double-Blind, Placebo-Controlled Study. World J Surg. 2015;39(11):2776-83.<br>• Kotzampassi K, Stavrou G, Damoraki G, Georgitsi M, Basdanis G, Tsaousi G, Giamarellos-Bourboulis E.J. Randomized, double-blind, placebo-controlled study of the efficacy of four probiotics to modify the risk for postoperative complications in colorectal surgery. Critical Care 2015 19 (S137-S138) SUPPL. 1
Sadahiro 2014 [30]	* Sadahiro S, Suzuki T, Tanaka A, Okada K, Kamata H, Ozaki T, et al. Comparison between oral antibiotics and probiotics as bowel preparation for elective colon cancer surgery to prevent infection: prospective randomized trial. Surgery. 2014;155(3):493-503.<br>• Influence of bifidobacterium on surgical site infection and enterobacterial flora in colorectal cancer surgery. https://center6.umin.ac.jp/cgi-open-bin/ctr/ctr_view.cgi?recptno=R000004159
Huanlongqin 2014 [31]	* Chen Hongqi XY, Shi Chenzhang, Liang Yong, Yang Yongzhi, Qin Huanlong. Effects of perioperative probiotics administration on patients with colorectal cancer. Chinese Journal of Clinical Nutrition. 2014;22:74-80.
Pellino 2013 [32]	* Pellino G, Sciaudone G, Candilio G, Camerlingo A, Marcellinaro R, De Fatico S, et al. Early postoperative administration of probiotics versus placebo in elderly patients undergoing elective colorectal surgery: a double-blind randomized controlled trial. BMC Surg. 2013;13 Suppl 2(Suppl 2):S57.
Liu 2013 [33]	* Liu ZH, Huang MJ, Zhang XW, Wang L, Huang NQ, Peng H, et al. The effects of perioperative probiotic treatment on serum zonulin concentration and subsequent postoperative infectious complications after colorectal cancer surgery: a double-center and double-blind randomized clinical trial. Am J Clin Nutr. 2013;97(1):117-26.<br>• Z. Liu, H. Qin, Z. Yang, Y. Xia, W. Liu, J. Yang, et al. Randomised clinical trial: the effects of perioperative probiotic treatment on barrier function and post-operative infectious complications in colorectal cancer surgery – a double-blind study. Alimentary Pharmacology and Therapeutics 2011; 33: 50–63<br>• Perioperative probiotics to improve gut barrier function and reduce postoperative infectious complications after colorectal cancer surgery: a randomized, double-blind controlled clinical trial. https://trialsearch.who.int/Trial2.aspx?TrialID=ChiCTR-TRC-09000423

Zhang 2012 [34]	* Zhang JW, Du P, Gao J, Yang BR, Fang WJ, Ying CM. Preoperative probiotics decrease postoperative infectious complications of colorectal cancer. Am J Med Sci. 2012;343(3):199-205.
Horvat 2010 [37]	* Horvat M, Krebs B, Potrc S, Ivanecz A, Kompan L. Preoperative synbiotic bowel conditioning for elective colorectal surgery. Wien Klin Wochenschr. 2010;122 Suppl 2:26-30.
Xia Yang 2010 [36]	* Xia Yang YZ, Chen Hong-qi, QIN Huan-long. Effect of bowel preparation with probiotics on intestinal barrier after surgery for colorectal cancer. Chinese Journal of Gastriintestinal Surgery. 2010;13:528-31.
Zhang 2010 [35]	* Zhang JW, Du P, Chen DW, Cui L, Ying CM. Effect of viable Bifidobacterium supplement on the immune status and inflammatory response in patients undergoing resection for colorectal cancer. Zhonghua Wei Chang Wai Ke Za Zhi. 2010;13(1):40-3.
Gianotti 2010 [38]	* Gianotti L, Morelli L, Galbiati F, Rocchetti S, Coppola S, Beneduce A, et al. A randomized double-blind trial on perioperative administration of probiotics in colorectal cancer patients. World J Gastroenterol. 2010;16(2):167-75.<br>• Erratum: A randomized double-blind trial on perioperative administration of probiotics in colorectal cancer patients (Journal of Gastrointestinal Cancer (2010) 16:2 (167-175))<br>• Probiotics In Colorectal Cancer Patients. https://www.clinicaltrials.gov/ct2/show/NCT00936572<br>• Gianotti, L.; Braga, M.; Morelli, L.; Galbiati, F.; Rocchetti, S.; Coppola, S.; Beneduce, A.; Gilardini, C.; Zonenschain, D.; Nespoli, A. Role of probiotics in colorectal surgery: A prospective randomized double-blind pilot study. Nutritional Therapy and Metabolism – 2008. Volume 26, Issue 4, pp. 190-198,

* This is the primary reference for the study

B. Park 2020 [24]

Study characteristics	
Methods	**Study design:** Prospective randomized controlled, double blinded parallel<br>**Setting/country:** multi-center (6)/Korea<br>**Date when study was conducted:** from December 2016 to June 2018
Participants	**Inclusion criteria**<br>• Histologically confirmed adenocarcinoma of the sigmoid colon, and indication for anterior resection.

	• Age (18–75 years)<br><br>**Exclusion criteria**<br>• Use of antibiotics or probiotics in the week before eligibility screening<br>• Pre-existing fecal or urinary incontinence<br>• Metastatic Colorectal cancer<br>• Preoperative endoscopic or symptomatic obstruction<br>• Severe cerebrovascular or heart disease,<br>• Pregnancy or lactation<br>• Inflammatory bowel disease<br>• History of chemotherapy or radiotherapy<br>• Symptoms of infectious or immunologic disease<br>• Abnormal creatinine (two-fold greater than the normal range), abnormal aspartate/alanine aminotransferase (three-fold greater than the normal range)<br>• Uncontrolled hypertension or diabetes, and refusal to participate.<br><br>**Total number of participants randomly assigned: 68**<br><br>**Probiotics**<br>• Number of all participant randomly assigned: 33<br>**Control**<br>• Number of all participant randomly assigned: 35
Interventions	**Intervention:** probiotics<br>**Comparator:** placebo<br>**Follow-up:** 1 month
Outcomes	**Outcomes**<br>• Anterior resection syndrome (ARS) quiestionnaire<br>• Changes in fecal microbiota,<br>• Inflammatory markers<br>• Nutritional screening index<br>• Quality of life (QoL) using European Organization for Research and Treatment of Cancer Quality-of-Life Questionnaire Core<br>• Postoperative complications
Funding sources	Korea Yakult Co., Ltd.
Declarations of interest	None
Notes	**Protocol:** NCT03531606<br>**Language of publication:** English

Risk of bias		
Bias	Authors' judgement	Support for judgement
Random sequence generation (selection bias)	Low risk	Quote: "the randomization table, a sequence of random numbers generated by the randomization program beginning with clinical trial subject number 1."
Allocation concealment (selection bias)	Unclear risk	Quote: "sequence concealed from investigators"
Blinding of participants and personnel (performance bias)	Low risk	Quote: "both patients and investigators were blinded to the assignments to the two

: All outcomes		groups"
Blinding of outcome assessment (detection bias)<br>: Subjective outcomes	Low risk	Quote: "both patients and investigators were blinded to the assignments to the two groups"
Blinding of outcome assessment (detection bias)<br>: Objective outcomes	Low risk	Judgement: objective outcomes were unlikely affected by lack of blinding
Incomplete outcome data (attrition bias)<br>: Perioperative mortality	Unclear risk	Judgement: No information (not measured)
Incomplete outcome data (attrition bias)<br>: Postoperative infectious complication	Low risk	Judgement: all participants were included in the analysis
Incomplete outcome data (attrition bias)<br>: Probiotics related adverse events	Low risk	Judgement: all participants were included in the analysis
Incomplete outcome data (attrition bias)<br>: Postoperative overall complication	Low risk	Judgement: all participants were included in the analysis
Incomplete outcome data (attrition bias)<br>: Hospital length of stay	Unclear risk	Judgement: No information (not measured)
Incomplete outcome data (attrition bias)<br>: Postoperative quality of life	Unclear risk	Judgement: in published article, there were only comments as follow “No difference in QOL and nutritional screening index was noted between the groups”
Selective reporting (reporting bias)	High risk	Judgement: study outcomes were not identical to the outcomes prespecified in the protocol
Other source of bias	Low risk	Judgement: not detected

QOL, quality of life;

C. Polakowskin 2019 [14]

Study characteristics	
Methods	**Study design:** prospective, randomized, double-blind, placebo-controlled study<br>**Setting/country:** single center/Brazil<br>**Date when study was conducted:** Not described
Participants	**Inclusion criteria**

	• Patients with CRC and underwent surgery as first-line treatment<br>**Exclusion criteria**<br>• Patients who were immunosuppressed<br>• Patients with metastatic disease<br>• Patients on enteral nutrition therapy<br>• Presence of unresectable tumors<br>• Presence of systemic infections requiring treatment with antibiotics in the previous 30 days<br>• Pregnant or breastfeeding women<br>• Patients who used prebiotics, probiotics, or synbiotics in the 3 months before the intervention<br>• Patients who were lost to follow-up<br><br>**Total number of participants randomly assigned: 73**<br><br>**Probiotics**<br>• Number of all participant randomly assigned: 36<br>**Control**<br>• Number of all participant randomly assigned: 37
Interventions	**Intervention:** Synbiotics<br>**Comparator:** Placebo<br>**Follow-up:** 1 month
Outcomes	• Serum albumin, transferrin, CRP, and IL-6<br>• Postoperative bowel function<br>• Occurrence of postoperative infections and or non-infectious complications<br>• Length of antibiotic usage<br>• Mortality in the period up to 30 days after the procedure
Funding sources	Not reported
Declarations of interest	None
Notes	**Protocol:** not identified<br>**Language of publication:** English

Risk of bias

Bias	Authors' judgement	Support for judgement
Random sequence generation (selection bias)	Low risk	Quote: "A randomizing sequence with two groups was generated by GraphPad statistical software"
Allocation concealment (selection bias)	Low risk	Quote: "Envelops containing either symbiotic or placebo-containing bags that were identical and sealed and packed in boxes labeled A or B were provided to study participants"
Blinding of participants and personnel (performance bias)<br>: All outcomes	Low risk	Quote: "double-blinded"
Blinding of outcome assessment (detection bias)<br>: Subjective outcomes	Low risk	Quote: "patients and study investigators remained blinded until after the statistical analysis was completed"
Blinding of outcome assessment	Low risk	Judgement: objective outcomes were unlikely

(detection bias)<br>: Objective outcomes		affected by lack of blinding.
Incomplete outcome data (attrition bias)<br>: Perioperative mortality	Low risk	Judgement: all participants were included in the analysis
Incomplete outcome data (attrition bias)<br>: Postoperative infectious complication	Low risk	Judgement: all participants were included in the analysis
Incomplete outcome data (attrition bias)<br>: Probiotics related adverse events	Low risk	Judgement: all participants were included in the analysis
Incomplete outcome data (attrition bias)<br>: Postoperative overall complication	Low risk	Judgement: all participants were included in the analysis
Incomplete outcome data (attrition bias)<br>: Hospital length of stay	Low risk	Judgement: all participants were included in the analysis
Incomplete outcome data (attrition bias)<br>: Postoperative quality of life	Unclear risk	Judgement: no information (not measured)
Selective reporting (reporting bias)	Unclear risk	Judgement: study protocol was not identified
Other source of bias	Low risk	Judgement: not detected

CRC, colorectal cancer; CRP, c-reactive protein; IL-6, interleukin-6

D. Bajramagic 2019 [25]

Study characteristics	
Methods	**Study design:** randomized controlled prospective study<br>**Setting/country:** single center/Bosnia<br>**Date when study was conducted:** from January 2017 to December 2017
Participants	**Inclusion criteria**<br>• Patients with colorectal adenocarcinoma<br>• ASA≤3, karnofsky index≥80%<br>• Without significant cardiac and neurological comorbidity<br>• Normal albumin level<br>• Stage III<br>• Patients who have undergone one of the standard surgical procedures with an open approach for the treatment of colorectal cancer<br>• Patients operated in the elective program

	**Exclusion criteria**<br>• not described<br><br>**Total number of participants randomly assigned: 78**<br><br>**Probiotics**<br>• Number of all participant randomly assigned: 39<br>**Control**<br>• Number of all participant randomly assigned: 39
Interventions	**Intervention:** Probiotics<br>**Comparator:** standard care (no placebo)<br>**Follow-up:** 1 year
Outcomes	• Postoperative complications<br>• SSI<br>• Anastomosis loosening<br>• Ileus<br>• Intraabdominal abscess
Funding sources	None
Declarations of interest	None
Notes	**Protocol:** not identified<br>**Language of publication:** English

Risk of bias

Bias	Authors' judgement	Support for judgement
Random sequence generation (selection bias)	Unclear risk	Judgement: not described
Allocation concealment (selection bias)	Unclear risk	Judgement: not described
Blinding of participants and personnel (performance bias)<br>: All outcomes	High risk	Judgement: The control group did not receive a placebo.
Blinding of outcome assessment (detection bias)<br>: Subjective outcomes	Unclear risk	Judgement: not described
Blinding of outcome assessment (detection bias)<br>: Objective outcomes	Low risk	Judgement: objective outcomes were unlikely affected by lack of blinding.
Incomplete outcome data (attrition bias)<br>: Perioperative mortality	Low risk	Judgement: all participants were included in the analysis
Incomplete outcome data (attrition bias)<br>: Postoperative infectious complication	Low risk	Judgement: all participants were included in the analysis
Incomplete outcome data (attrition bias)<br>: Probiotics related adverse events	Unclear risk	Judgement: no information (not measured)

Incomplete outcome data (attrition bias)<br>: Postoperative overall complication	Low risk	Judgement: all participants were included in the analysis
Incomplete outcome data (attrition bias)<br>: Hospital length of stay	Unclear risk	Judgement: no information (not measured)
Incomplete outcome data (attrition bias)<br>: Postoperative quality of life	Unclear risk	Judgement: no information (not measured)
Selective reporting (reporting bias)	Unclear risk	Judgement: study protocol was not identified
Other source of bias	Unclear risk	Judgement: There is no analysis about baseline characteristics

ASA, American society of anesthesiologists; SSI, surgical site infection

E. Flesche 2017 [26]

Study characteristics	
Methods	**Study design:** randomized, double-blinded, placebo-controlled trial<br>**Setting/country:** single center/Brazil<br>**Date when study was conducted:** from June 2013 to April 2015
Participants	**Inclusion criteria**<br>• Histologically proven colorectal adenocarcinoma with indication of elective and potentially curative colorectal resection<br>**Exclusion criteria**<br>• Pregnancy<br>• Patient's difficulties regarding adequate understanding of the study<br>• Neoadjuvant treatment (chemotherapy and radiotherapy)<br>• Previous use of products with prebiotic, probiotic and/or symbiotic function or fiber modulus<br>• Refusal to participate<br>• Patients with tumors that were considered non-resectable during surgery<br>• Those who had other organs resected concomitantly (uterus, bladder, liver, spleen)<br><br>**Total number of participants randomly assigned: 91**<br><br>**Probiotics**<br>• Number of all participant randomly assigned: 49<br>**Control**

	• Number of all participant randomly assigned: 42
Interventions	**Intervention:** Synbiotics<br>**Comparator:** Placebo<br>**Follow-up:** 1 month
Outcomes	**Primary outcome**<br>• Postoperative infection (incisional, organ/space, UTI, pneumonia)<br>**Secondary outcome**<br>• Length of hospital stay<br>• Deaths occurring within 30 days of surgery
Funding sources	None
Declarations of interest	None
Notes	**Protocol:** NCT01468779<br>**Language of publication:** English

Risk of bias		
Bias	Authors' judgement	Support for judgement
Random sequence generation (selection bias)	Low risk	Quote: "computer-generated sequence of numbers"
Allocation concealment (selection bias)	Unclear risk	Judgement: not described
Blinding of participants and personnel (performance bias)<br>: All outcomes	Low risk	Quote: "both the investigators and the patients were blinded as to group allocation until the end of the test"
Blinding of outcome assessment (detection bias)<br>: Subjective outcomes	Low risk	Quote: "both the investigators and the patients were blinded as to group allocation until the end of the test"
Blinding of outcome assessment (detection bias)<br>: Objective outcomes	Low risk	Judgement: objective outcomes were unlikely affected by lack of blinding.
Incomplete outcome data (attrition bias)<br>: Perioperative mortality	Unclear risk	Quote: only mentioned as follow "there were no significant differences between the groups regarding mortality rates"
Incomplete outcome data (attrition bias)<br>: Postoperative infectious complication	Unclear risk	Quote: only mentioned as follow "only one patient in the symbiotics group presented surgical wound infection, while nine cases were diagnosed in the control group"
Incomplete outcome data (attrition bias)<br>: Probiotics related adverse events	Unclear risk	Judgement: no information (not measured)
Incomplete outcome data (attrition bias)<br>: Postoperative overall complication	Unclear risk	Quote: only mentioned as follow "only one patient in the symbiotics group presented surgical wound infection, while nine cases were diagnosed in the control group" " the incidence of non-infectious postoperative complications such as nausea, vomiting, abdominal distension, ileus, diarrhea, or constipation was not different between the study groups"

Incomplete outcome data (attrition bias)<br>: Hospital length of stay	Unclear risk	Quote: only mentioned as follow "the mean hospitalization time was 11.2 days for the patients in the symbiotics group and 12.69 days for the patients in the control group, with no statistical significance"
Incomplete outcome data (attrition bias)<br>: Postoperative quality of life	Unclear risk	Judgement: no information (not measured)
Selective reporting (reporting bias)	Low risk	Judgement: not detected
Other source of bias	High risk	Judgement: The study protocol and the contents of the published journal are completely different. (The study subjective was periampullary cancer in the protocol, while the study subjective was colorectal cancer in the full-text article)

UTI, urinary tract infection

F. Yang 2016 [15]

Study characteristics	
Methods	**Study design:** Prospective randomized controlled<br>**Setting/country:** single center/China<br>**Date when study was conducted:** from November 2011 to September 2012
Participants	**Inclusion criteria**<br>• Patients who diagnosed with sporadic CRC<br>• Age 25 to 80<br>• No evidence of cancer metastasis<br>• Joined the trial voluntarily and provided an informed consent<br>**Exclusion criteria**<br>• Age more than 80 or younger than 25<br>• Co-occurrence of other GI disease<br>• Co-existence of other malignant neoplasm<br>• Severe cardiovascular and cerebrovascular disease that could not tolerate radical surgery<br>• Distant metastasis<br>• Recent use of probiotics, synbiotics<br>• Recent infection or antibiotics use<br>• Emergency surgery<br>• Laparoscopic surgery<br>• Neoadjuvant CRT<br>• Immunodeficiency<br>• Pregnancy<br><br>**Total number of participants randomly assigned: 79**

	**Probiotics**<br>• Number of all participant randomly assigned: 42<br>**Control**<br>• Number of all participant randomly assigned: 37
Interventions	**Intervention:** Probiotics<br>**Comparator:** Placebo<br>**Follow-up:** 1 month
Outcomes	**Primary outcome**<br>• Influence of the diarrhea<br>**Secondary outcome**<br>• The influence of the bowel function (days to first flatus, defecation)<br>• The incidence of the other complications<br>• Rate of non-infectious complications, infectious complications (bacteremia, wound infection, pneumonia, urinary tract infection, anastomotic leakage, abdominal distension)<br>• postoperative hospital stay<br>• Mortality
Funding sources	National Natural Science Foundation of China (No.81230057; No.81472262; No.81372615), the National High Technology Research and Development Program (863 Program; Grant No. 2014AA020803), the National Science and Technology Major Projects (2013ZX09103003-16), Shanghai Health System Outstanding<br>Young Talent Training Plan (No.XYQ2013118)
Declarations of interest	None
Notes	**Protocol:** ChiCTR-TRC-13003332<br>**Language of publication:** English

Risk of bias

Bias	Authors' judgement	Support for judgement
Random sequence generation (selection bias)	Low risk	Quote: "random number table from protocol"
Allocation concealment (selection bias)	Unclear risk	Judgement: not described
Blinding of participants and personnel (performance bias)<br>: All outcomes	Low risk	Quote: "All researchers and subjects were blinded to randomization and treatments during the entire interventional period"
Blinding of outcome assessment (detection bias)<br>: Subjective outcomes	Low risk	Quote: "All researchers and subjects were blinded to randomization and treatments during the entire interventional period"
Blinding of outcome assessment (detection bias)<br>: Objective outcomes	Low risk	Judgement: objective outcomes were unlikely affected by lack of blinding.
Incomplete outcome data (attrition bias)<br>: Perioperative mortality	Low risk	Judgement: all participants were included in the analysis
Incomplete outcome data (attrition bias)<br>: Postoperative infectious complication	Low risk	Judgement: all participants were included in the analysis

Incomplete outcome data (attrition bias)<br>: Probiotics related adverse events	Low risk	Judgement: all participants were included in the analysis
Incomplete outcome data (attrition bias)<br>: Postoperative overall complication	Low risk	Judgement: all participants were included in the analysis
Incomplete outcome data (attrition bias)<br>: Hospital length of stay	Low risk	Judgement: all participants were included in the analysis
Incomplete outcome data (attrition bias)<br>: Postoperative quality of life	Unclear risk	Judgement: No information (not measured)
Selective reporting (reporting bias)	High risk	Judgement: The outcomes in protocol were different from the full text article
Other source of bias	Low risk	Judgement: not detected

CRC, colorectal cancer; GI, gastrointestinal; CRT, chemoradiation therapy

G. Theodoropoulos 2016 [27]

Study characteristics	
Methods	**Study design:** prospective, double-blind, randomized control trial<br>**Setting/country:** single center/Japan<br>**Date when study was conducted:** from July 2008 to April 2012
Participants	**Inclusion criteria**<br>• Patients with histological documentation of cancer of the colon or rectum<br>• Aged between 18 and 80 years<br>• Candidates for elective colorectal resection for cancer<br>**Exclusion criteria**<br>• Denied written informed consent<br>• Pregnant<br>• Hereditary cancer<br>• History of inflammatory bowel disease (IBD)<br>• Metastatic disease at presentation<br>• Required permanent or temporary stoma<br>• Emergency operation<br>• Major postoperative complications<br>• Had disease progression during the study period<br>• Did not tolerate liquid diet by the 5th postoperative day (POD).<br><br>**Total number of participants randomly assigned: 75**

	**Probiotics**<br>• Number of all participant randomly assigned: 38<br>**Control**<br>• Number of all participant randomly assigned: 37
Interventions	**Intervention:** Synbiotics<br>**Comparator:** Placebo<br>**Follow-up:** 6 months
Outcomes	**Outcomes**<br>• Gastrointestinal function-related quality of life (GIQLI)<br>• Assessment of functional bowel disorders (constipation, diarrhea)
Funding sources	Not reported
Declarations of interest	None
Notes	**Protocol:** NCT01479907<br>**Language of publication:** English

Risk of bias

Bias	Authors' judgement	Support for judgement
Random sequence generation (selection bias)	Low risk	Quote: "computer-generated random allocation schedule"
Allocation concealment (selection bias)	Low risk	Quote: "sequentially numbered sealed opaque envelopes technique"
Blinding of participants and personnel (performance bias)<br>: All outcomes	Low risk	Quote: "the identity of the specific product was blind to participants, support staff and investigators"
Blinding of outcome assessment (detection bias)<br>: Subjective outcomes	Low risk	Quote: "Quadruple (Participant, Care Provider, Investigator, Outcomes Assessor)"
Blinding of outcome assessment (detection bias)<br>: Objective outcomes	Low risk	Judgement: objective outcomes were unlikely affected by lack of blinding.
Incomplete outcome data (attrition bias)<br>: Perioperative mortality	Unclear risk	Judgement: No information (not measured)
Incomplete outcome data (attrition bias)<br>: Postoperative infectious complication	Unclear risk	Judgement: No information (not measured)
Incomplete outcome data (attrition bias)<br>: Probiotics related adverse events	Unclear risk	Judgement: No information (not measured)
Incomplete outcome data (attrition bias)<br>: Postoperative overall complication	Unclear risk	Judgement: No information (not measured)
Incomplete outcome data (attrition bias)<br>: Hospital length of stay	Unclear risk	Judgement: No information (not measured)

Incomplete outcome data (attrition bias)<br>: Postoperative quality of life	Unclear risk	Judgement: No information (not measured)
Selective reporting (reporting bias)	Low risk	Judgement: not detected
Other source of bias	Low risk	Judgement: not detected

IBD, inflammatory bowel disease; POD, postoperative day; GIQLI, Gastrointestinal function-related quality of life

H. Tan 2016 [16]

Study characteristics	
Methods	**Study design:** randomized, double-blind, placebo controlled trial<br>**Setting/country:** single center/Malaysia<br>**Date when study was conducted:** from June 2012 to May 2015
Participants	**Inclusion criteria**<br>• Aged 18 years and above<br>• Diagnosed with colorectal cancer, and scheduled for surgery<br>**Exclusion criteria**<br>• Acute intestinal obstruction, immunodeficiency<br>• Evidence of preexisting infection<br>• Emergency surgery<br>• Inability to tolerate regular oral ingestion of probiotics within 1 week prior to recruitment<br><br>**Total number of participants randomly assigned: 40**<br><br>**Probiotics**<br>• Number of all participant randomly assigned: 20<br>**Control**<br>• Number of all participant randomly assigned: 20
Interventions	**Intervention:** Probiotics<br>**Comparator:** Placebo<br>**Follow-up:** 1 month
Outcomes	**Primary outcomes**<br>• Time to return of normal gut function<br>**Secondary outcomes**<br>• The duration of hospital stay<br>• Postoperative complications (wound infection, pneumonia, anastomotic leak, line infection, UTI, DVT, renal failure, liver failure, respiratory failure, hematologic failure mortality)<br>• Postoperative/paralytic ileus,<br>• Abdominal discomfort and pain

	• Early mobilisation and oral intake assessment
Funding sources	B-Crobes Laboratory Sdn. Bhd
Declarations of interest	None
Notes	**Protocol:** ACTRN12615000545561<br>**Language of publication:** English

Risk of bias

Bias	Authors' judgement	Support for judgement
Random sequence generation (selection bias)	Low risk	Quote: "Stat Trek's random number generator, which uses a statistical algorithm to produce random numbers, was used to randomize subjects into the treatment and placebo groups"
Allocation concealment (selection bias)	Unclear risk	Judgement: not described
Blinding of participants and personnel (performance bias)<br>: All outcomes	Low risk	Quote: "blinded (patients, administrator, outcome assessor, analyzer)"
Blinding of outcome assessment (detection bias)<br>: Subjective outcomes	Low risk	Quote: "blinded (patients, administrator, outcome assessor, analyzer)"
Blinding of outcome assessment (detection bias)<br>: Objective outcomes	Low risk	Judgement: objective outcomes were unlikely affected by lack of blinding.
Incomplete outcome data (attrition bias)<br>: Perioperative mortality	Low risk	Judgement: all participants were included in the analysis
Incomplete outcome data (attrition bias)<br>: Postoperative infectious complication	Low risk	Judgement: all participants were included in the analysis
Incomplete outcome data (attrition bias)<br>: Probiotics related adverse events	Unclear risk	Judgement: No information (not measured)
Incomplete outcome data (attrition bias)<br>: Postoperative overall complication	Low risk	Judgement: all participants were included in the analysis
Incomplete outcome data (attrition bias)<br>: Hospital length of stay	Low risk	Judgement: all participants were included in the analysis
Incomplete outcome data (attrition bias)<br>: Postoperative quality of life	Unclear risk	Judgement: No information (not measured)
Selective reporting (reporting bias)	High risk	Judgement: Postoperative ileus was included in the outcome specified in the study protocol, but there is no mention of the outcome of ileus in the full text article.

Other source of bias	Low risk	Judgement: not detected

UTI, urinary tract infection; DVT, deep vein thrombosis

I. Mizuta 2016 [28]

Study characteristics	
Methods	**Study design:** randomized, single-center, single-blinded trial<br>**Setting/country:** single center/Japan<br>**Date when study was conducted:** from October 2008 to December 2012
Participants	**Inclusion criteria**<br>• Patients who had colorectal cancer and were scheduled to undergo colorectal cancer resection<br>• Age at 20-85 years<br>**Exclusion criteria**<br>• Severe diseases of the liver, kidney, heart, and lung<br>• Presence of a food allergy<br>• Patients with bowel obstruction<br><br>**Total number of participants randomly assigned:** 60<br><br>**Probiotics**<br>• Number of all participant randomly assigned: 31<br>**Control**<br>• Number of all participant randomly assigned: 29
Interventions	**Intervention:** Probiotics<br>**Comparator:** standard care (no placebo)<br>**Follow-up:** 2 weeks
Outcomes	• Postoperative complications (infectious and non-infectious, hospital stay, superficial, deep infection, anastomotic leak)<br>• Serologic marker (WBC, erythrocytes, Hb, lymphocytes, platelets, total protein, albumin, CRP, IL-6, natural killer cell)<br>• Fecal microbiota
Funding sources	Not reported
Declarations of interest	None
Notes	**Protocol:** not identified<br>**Language of publication:** English
Risk of bias	

Bias	Authors' judgement	Support for judgement

Random sequence generation (selection bias)	Low risk	Quote: "computer generated permuted block randomization"
Allocation concealment (selection bias)	Unclear risk	Judgement: not described
Blinding of participants and personnel (performance bias)<br>: All outcomes	High risk	Quote: "single blinded, physicians involved with the diagnosis of infections and researchers performing the statistical analyses were not involved in the randomization"
Blinding of outcome assessment (detection bias)<br>: Subjective outcomes	Low risk	Quote: "physicians involved with the diagnosis of infections and researchers performing the statistical analyses were not involved in the randomization"
Blinding of outcome assessment (detection bias)<br>: Objective outcomes	Low risk	Judgement: objective outcomes were unlikely affected by lack of blinding.
Incomplete outcome data (attrition bias)<br>: Perioperative mortality	Unclear risk	Judgement: No information (not measured)
Incomplete outcome data (attrition bias)<br>: Postoperative infectious complication	Low risk	Judgement: all participants were included in the analysis
Incomplete outcome data (attrition bias)<br>: Probiotics related adverse events	Unclear risk	Judgement: No information (not measured)
Incomplete outcome data (attrition bias)<br>: Postoperative overall complication	Low risk	Judgement: all participants were included in the analysis
Incomplete outcome data (attrition bias)<br>: Hospital length of stay	Low risk	Judgement: all participants were included in the analysis
Incomplete outcome data (attrition bias)<br>: Postoperative quality of life	Unclear risk	Judgement: No information (not measured)
Selective reporting (reporting bias)	Unclear risk	Judgement: study protocol was not identified
Other source of bias	Low risk	Judgement: not detected

WBC, white blood cell; Hb, hemoglobin; CRP, C-reactive protein; IL-6, interleukin-6

J. Krebs 2016 [29]

Study characteristics	
Methods	**Study design:** prospective randomized, controlled, double blind trial of three groups<br>**Setting/country:** single center/Slovenia<br>**Date when study was conducted:** from December 2009 to December 2012
Participants	**Inclusion criteria**<br>• Patients with preceding large bowel operation for colorectal cancer<br>**Exclusion criteria**<br>• Any chronic disease that may interfere with the patient's ability to comply with protocol<br>• Bowel obstruction patients who could not take the mechanic preoperative bowel preparation<br>• Patients with chronic inflammatory bowel disease<br>• Non-compliance, refusal of the patient to continue treatment<br><br>**Total number of participants randomly assigned:** 54<br><br>**Probiotics**<br>• Number of all participant randomly assigned: 18<br>**Prebiotics**<br>• Number of all participant randomly assigned: 20<br>**Control**<br>• Number of all participant randomly assigned: 16
Interventions	**Intervention:** Synbiotics<br>**Comparator:** standard care (no placebo)<br>**Follow-up:** 1 month
Outcomes	**Outcomes**<br>• The number of four different probiotic bacteria on colonic mucosa<br>• Serologic biomarker (CRP, IL-6, fibrinogen, leukocyte count, differential blood count)<br>• First peristalsis<br>• First solid oral food intake<br>• Passing of gasses<br>• Passing of stool<br>• Hospital length of stay<br>• Postoperative complications (bowel paresis, wound infection, pneumonia)
Funding sources	None
Declarations of interest	None
Notes	**Protocol:** Not identified<br>**Language of publication:** English

Risk of bias		
Bias	Authors' judgement	Support for judgement
Random sequence generation (selection bias)	Unclear risk	Judgement: not described

Allocation concealment (selection bias)	Low risk	Quote: "sealed opaque envelopes"
Blinding of participants and personnel (performance bias)<br>: All outcomes	High risk	Judgement: The identity of the product was not known to participants or researchers until the end of the study, however, the control group did not receive a placebo.
Blinding of outcome assessment (detection bias)<br>: Subjective outcomes	Unclear risk	Judgement: The identity of the product was not known to participants or researchers until the end of the study, however, the control group did not receive a placebo.
Blinding of outcome assessment (detection bias)<br>: Objective outcomes	Low risk	Judgement: objective outcomes were unlikely affected by lack of blinding.
Incomplete outcome data (attrition bias)<br>: Perioperative mortality	Unclear risk	Judgement: no information (not measured)
Incomplete outcome data (attrition bias)<br>: Postoperative infectious complication	Unclear risk	Judgement: "9 patients had postoperative bowel paresis, four patients had wound infection and two pneumonia. All complications were treated conservatively.
Incomplete outcome data (attrition bias)<br>: Probiotics related adverse events	Unclear risk	Judgement: There is an information, but no information how many randomized people were loose
Incomplete outcome data (attrition bias)<br>: Postoperative overall complication	Unclear risk	Judgement: "9 patients had postoperative bowel paresis, four patients had wound infection and two pneumonia. All complications were treated conservatively.
Incomplete outcome data (attrition bias)<br>: Hospital length of stay	Low risk	Judgement: all participants were included in the analysis
Incomplete outcome data (attrition bias)<br>: Postoperative quality of life	Unclear risk	Judgement: No information (not measured)
Selective reporting (reporting bias)	Unclear risk	Judgement: study protocol was not identified
Other source of bias	Unclear risk	Judgement: discrepancy between 3 groups was not shown

CRP, C-reactive protein; IL-6, interleukin-6

K. Komatsu 2016 [17]

Study characteristics	
Methods	**Study design:** randomized controlled trial<br>**Setting/country:** single center/Japan<br>**Date when study was conducted:** from June 2008 to December 2013
Participants	**Inclusion criteria**<br>• Patients scheduled to undergo elective laparoscopic colorectal surgery<br>**Exclusion criteria**<br>• The need for additional surgical treatments (such as hepatectomy or urinary tract diversion)<br>• Current routine use of probiotics or synbiotics<br>• Preferring or disliking taking probiotics or synbiotics<br>• Difficulty in drinking water due to conditions (such as bowel obstruction)<br>• Schedule that did not permit synbiotics treatment for more than 7 days before surgery<br>• Lack of understanding of the study's concept<br><br>**Total number of participants randomly assigned:** 379<br><br>**Probiotics**<br>• Number of all participant randomly assigned: 173<br>**Control**<br>• Number of all participant randomly assigned: 206
Interventions	**Intervention:** Synbiotics<br>**Comparator:** standard care (no placebo)<br>**Follow-up:** 1 month
Outcomes	**Primary outcomes**<br>• Postoperative infectious complications (SSI, anastomotic leak, ileus, MRSA infection, C.difficile infection, non-surgical site infection, reoperation, readmission, mortality)<br>**Secondary outcomes**<br>• Fecal microflora, fecal organic acid concentration
Funding sources	Yakult Central Institute for Microbiological Research
Declarations of interest	None
Notes	**Protocol:** UMIN000003439<br>**Language of publication:** English

Risk of bias		
Bias	Authors' judgement	Support for judgement
Random sequence generation (selection bias)	Low risk	Quote: "computer-based randomization, FileMaker Pro"
Allocation concealment (selection bias)	Low risk	Quote: "complete concealment of the randomization sequence"
Blinding of participants and personnel (performance bias)<br>: All outcomes	High risk	Quote: "the patients were not blinded to which group they were in, and no placebo product was used"
Blinding of outcome assessment (detection bias)<br>: Subjective outcomes	Unclear risk	Judgement: not described
Blinding of outcome assessment	Low risk	Judgement: objective outcomes were unlikely

(detection bias)<br>: Objective outcomes		affected by lack of blinding.
Incomplete outcome data (attrition bias)<br>: Perioperative mortality	Low risk	Judgement: all participants were included in the analysis
Incomplete outcome data (attrition bias)<br>: Postoperative infectious complication	Low risk	Judgement: all participants were included in the analysis
Incomplete outcome data (attrition bias)<br>: Probiotics related adverse events	Low risk	Judgement: all participants were included in the analysis
Incomplete outcome data (attrition bias)<br>: Postoperative overall complication	Low risk	Judgement: all participants were included in the analysis
Incomplete outcome data (attrition bias)<br>: Hospital length of stay	Unclear risk	Judgement: no information (not measured)
Incomplete outcome data (attrition bias)<br>: Postoperative quality of life	Unclear risk	Judgement: No information (not measured)
Selective reporting (reporting bias)	High risk	Judgement: Outcomes in the protocol were different from outcomes in the article
Other source of bias	Low risk	Judgement: Not detected

SSI, surgical site infection; MRSA, Methicillin-resistant Staphylococcus aureus

L. Kotzampassi 2016 [18]

Study characteristics	
Methods	**Study design:** randomized, double-blind, placebo-controlled trial<br>**Setting/country:** single center/Greece<br>**Date when study was conducted:** from April 2013 to July 2014
Participants	**Inclusion criteria**<br>• both gender

	• Age≥18 years old’<br>• Acceptable nutritional status (i.e. serum albumin > 3.5gr/dL, NRS 2002 score≤3)<br>• Programmed for open surgery for colorectal cancer<br>**Exclusion criteria**<br>• Age<18 years<br>• Denial or inability to consent<br>• Need for emergency or palliative surgery<br>• American Society of Anesthesiologists (ASA) class IV or V<br>• Pregnancy or lactation<br>• Inflammatory bowel disease<br>• Use of antibiotics the last 10 days before surgery<br>• Recent steroid therapy or preoperative neoadjuvant chemotherapy or radiotherapy<br>• Signs of bacterial infection (defined by white cell count and body temperature)<br>• Infection by hepatitis B or C virus by human immunodeficiency virus and by cytomegalovirus<br><br>**Total number of participants randomly assigned:** 168<br><br>**Probiotics**<br>• Number of all participant randomly assigned: 86<br>**Control**<br>• Number of all participant randomly assigned: 82
Interventions	**Intervention:** Probiotics<br>**Comparator:** Placebo<br>**Follow-up:** 1 month
Outcomes	**Outcomes**<br>• Occurrence of major postoperative complications within 30 days (anastomotic leakage, abdominal wound infection and dehiscence, and any infection accompanied or not by severe sepsis)<br>• Occurrence of minor postoperative complications within 30 days (peripheral vein thrombosis, pulmonary embolism, acute heart failure and acute renal failure)<br>• The time until development of complications within 30 days<br>• Days on mechanical ventilation<br>• Duration of postoperative ileus and total hospital days<br>• Gene expression and serum cytokines (SOCS3, TNF-α, IL-6)
Funding sources	UniPharma, Athens, Greece
Declarations of interest	None
Notes	**Protocol:** NCT02313519<br>**Language of publication:** English

Risk of bias

Bias	Authors’ judgement	Support for judgement
Random sequence generation (selection bias)	Low risk	Quote: "The allocated sequence was prepared at a 1:1 ratio by a biostatistician"
Allocation concealment	Unclear risk	Quote: "The patients were randomized by the

(selection bias)		sealed envelope method"<br><br>Judgement: There was no mention about 'opaque'
Blinding of participants and personnel (performance bias)<br>: All outcomes	Low risk	Quote: "Both surgeons and physicians in carge, participants and study investigators were blinded to this randomization"
Blinding of outcome assessment (detection bias)<br>: Subjective outcomes	Low risk	Quote: "Both surgeons and physicians in carge, participants and study investigators were blinded to this randomization<br><br>masking triple (participant, care provider, investigator)"
Blinding of outcome assessment (detection bias)<br>: Objective outcomes	Low risk	Judgement: objective outcomes were unlikely affected by lack of blinding.
Incomplete outcome data (attrition bias)<br>: Perioperative mortality	Unclear risk	Judgement: no information (not measured)
Incomplete outcome data (attrition bias)<br>: Postoperative infectious complication	Low risk	Judgement: all participants were included in the analysis
Incomplete outcome data (attrition bias)<br>: Probiotics related adverse events	Unclear risk	Judgement: no information (not measured)
Incomplete outcome data (attrition bias)<br>: Postoperative overall complication	Low risk	Judgement: all participants were included in the analysis
Incomplete outcome data (attrition bias)<br>: Hospital length of stay	Unclear risk	Judgement: There is a KM curve, but the number of patients that used for the analysis was not identified
Incomplete outcome data (attrition bias)<br>: Postoperative quality of life	Unclear risk	Judgement: No information (not measured)
Selective reporting (reporting bias)	Low risk	Judgement: Not detected
Other source of bias	Low risk	Judgement: Not detected

NRS, nutrition risk screening ; ASA, American Society of Anesthesiologists ; SOCS3, Suppressor Of Cytokine Signaling 3 ; TNF-α, tumour necrosis factor-alpha ; IL-6, interleukin-6; KM curve, kaplan-meier curve

M. Sadahiro 2014 [30]

Study characteristics	
Methods	**Study design:** prospective randomized controlled trial<br>**Setting/country:** single center/Japan<br>**Date when study was conducted:** from May 2008 to October 2011
Participants	**Inclusion criteria**<br>• Patients scheduled to undergo elective colon cancer operations in whom curative resection of tumor<br>• 20 to 80 years of age<br>• Preoperative performance status of 0 or 1<br>• No serious coexisting medical conditions<br>**Exclusion criteria**<br>• History of intestinal resection<br>• Patients with a stoma<br>• Patients with intestinal stenosis or obstruction that would preclude routine preoperative mechanical bowel preparation<br>• Patients with stage IV disease on preoperative diagnosis<br><br>**Total number of participants randomly assigned:** 294<br><br>**Probiotics**<br>• Number of all participant randomly assigned: 100<br>**Antibiotics**<br>• Number of all participant randomly assigned: 99<br>**Control**<br>• Number of all participant randomly assigned: 95
Interventions	**Intervention:** Probiotics<br>**Comparator:** standard care (no placebo)<br>**Follow-up:** 1 month
Outcomes	**Outcomes**<br>• SSI (incisional or organ/space)<br>• Incidence of remote infection<br>• Anastomotic leakage<br>• CD toxin<br>• Change in the number of bacteria in feces
Funding sources	None
Declarations of interest	None
Notes	**Protocol:** UMIN000003435<br>**Language of publication:** English

Risk of bias		
Bias	Authors'	Support for judgement

	judgement	
Random sequence generation (selection bias)	Low risk	Quote: "Randomization was performed by one of the authors (H.K.) according to the minimization method with tumor site as a stratification factor"
Allocation concealment (selection bias)	Unclear risk	Judgement: not described
Blinding of participants and personnel (performance bias)<br>: All outcomes	High risk	Judgement: The control group did not receive a placebo.
Blinding of outcome assessment (detection bias)<br>: Subjective outcomes	Unclear risk	Judgement: not described
Blinding of outcome assessment (detection bias)<br>: Objective outcomes	Low risk	Judgement: objective outcomes were unlikely affected by lack of blinding.
Incomplete outcome data (attrition bias)<br>: Perioperative mortality	Unclear risk	Judgement: no information (not measured)
Incomplete outcome data (attrition bias)<br>: Postoperative infectious complication	Low risk	Judgement: all participants were included in the analysis
Incomplete outcome data (attrition bias)<br>: Probiotics related adverse events	Unclear risk	Judgement: no information (not measured)
Incomplete outcome data (attrition bias)<br>: Postoperative overall complication	Low risk	Judgement: all participants were included in the analysis
Incomplete outcome data (attrition bias)<br>: Hospital length of stay	Unclear risk	Judgement: No information (not measured)
Incomplete outcome data (attrition bias)<br>: Postoperative quality of life	Unclear risk	Judgement: No information (not measured)
Selective reporting (reporting bias)	Low risk	Judgement: not detected
Other source of bias	Low risk	Judgement: not detected

SSI, surgical site infection; CD toxin, clostridium difficile toxin

N. Huanlongqin 2014 [31]

Study characteristics	
Methods	**Study design:** prospective randomized controlled trial<br>**Setting/country:** single center/China<br>**Date when study was conducted:** from May 2011 to July 2011
Participants	**Inclusion criteria**<br>• Patients with elective surgery for CRC who were confirmed to be colorectal cancer by colonoscopy and biopsy before surgery<br>• Operable case<br>**Exclusion criteria**<br>• Metastatic colorectal cancer<br>• Inoperable<br>• Obstruction, perforation, bleeding<br>• Severe anemia (Hb<6), severe liver, kidney, cardiovascular and cerebrovascular chronic disease, chronic severe metabolic disease<br>• Preop RT or CTx<br>• Refusal<br>• Infectious general status<br><br>**Total number of participants randomly assigned:** 70<br><br>**Probiotics**<br>• Number of all participant randomly assigned: 35<br>**Control**<br>• Number of all participant randomly assigned: 35
Interventions	**Intervention:** Probiotics<br>**Comparator:** Placebo<br>**Follow-up:** 1 month
Outcomes	**Outcomes**<br>• Intestinal epithelial junction and permeability<br>• Fecal microflora<br>• First exhaust and defecation time<br>• Abdominal distension and diarrhea incidence<br>• Inflammatory response<br>• Postoperative infective complications (SSI, UTI, pneumonia)"
Funding sources	Not reported
Declarations of interest	None
Notes	**Protocol:** Not identified<br>**Language of publication:** Chinese

Risk of bias		
Bias	Authors' judgement	Support for judgement
Random sequence generation (selection bias)	Low risk	Quote: "random number table method"
Allocation concealment	Unclear risk	Judgement: not described

(selection bias)		
Blinding of participants and personnel (performance bias)<br>: All outcomes	Low risk	Quote: "the outer packing of the two is exactly the same"
Blinding of outcome assessment (detection bias)<br>: Subjective outcomes	Unclear risk	Judgement: not described
Blinding of outcome assessment (detection bias)<br>: Objective outcomes	Low risk	Judgement: objective outcomes were unlikely affected by lack of blinding.
Incomplete outcome data (attrition bias)<br>: Perioperative mortality	Unclear risk	Judgement: no information (not measured)
Incomplete outcome data (attrition bias)<br>: Postoperative infectious complication	Unclear risk	Judgement: No information (not measured)
Incomplete outcome data (attrition bias)<br>: Probiotics related adverse events	Low risk	Judgement: all participants were included in the analysis
Incomplete outcome data (attrition bias)<br>: Postoperative overall complication	Unclear risk	Judgement: no information (not measured)
Incomplete outcome data (attrition bias)<br>: Hospital length of stay	Low risk	Judgement: all participants were included in the analysis
Incomplete outcome data (attrition bias)<br>: Postoperative quality of life	Unclear risk	Judgement: No information (not measured)
Selective reporting (reporting bias)	Unclear risk	Judgement: study protocol was not identified
Other source of bias	Low risk	Judgement: Not detected

CRC, colorectal cancer; Hb, hemoglobin; RT, radiation therapy; CTx, chemotherapy; SSI, surgical site infection; UTI, urinary tract infection

O. Pellino 2013 [32]

Study characteristics	
Methods	**Study design:** prospective double blind randomized trial

	**Setting/country:** single center/Italy<br>**Date when study was conducted:** from 2005 to January 2012
Participants	**Inclusion criteria**<br>• Patients aged over 70 years undergoing laparoscopic colonic resection<br>**Exclusion criteria**<br>• Dukes D colorectal cancer<br>• Inflammatory bowel diseases<br>• Need for temporary or definitive ostomy<br>• Allergy to antibiotics/probiotics/placebo used in the study<br><br>**Total number of participants randomly assigned:** 18<br><br>**Probiotics**<br>• Number of all participant randomly assigned: 10<br>**Control**<br>• Number of all participant randomly assigned: 8
Interventions	**Intervention:** Probiotics<br>**Comparator:** Placebo<br>**Follow-up:** 1 month
Outcomes	**Outcomes**<br>• Bowel function<br>• Quality of life<br>• Postoperative complications<br>• Hospital length of stay
Funding sources	Personal funds
Declarations of interest	None
Notes	**Protocol:** Not identified<br>**Language of publication:** English

Risk of bias

Bias	Authors' judgement	Support for judgement
Random sequence generation (selection bias)	Unclear risk	Judgement: not described
Allocation concealment (selection bias)	Unclear risk	Judgement: not described
Blinding of participants and personnel (performance bias)<br>: All outcomes	Unclear risk	Judgement: not described
Blinding of outcome assessment (detection bias)<br>: Subjective outcomes	Unclear risk	Judgement: not described
Blinding of outcome assessment (detection bias)<br>: Objective outcomes	Low risk	Judgement: objective outcomes were unlikely affected by lack of blinding.
Incomplete outcome data (attrition bias)<br>: Perioperative mortality	Unclear risk	Judgement: no information (not measured)
Incomplete outcome data (attrition bias)	Unclear risk	Judgement: no information (not measured)

: Postoperative infectious complication		
Incomplete outcome data (attrition bias)<br>: Probiotics related adverse events	Unclear risk	Judgement: no information (not measured)
Incomplete outcome data (attrition bias)<br>: Postoperative overall complication	Unclear risk	Judgement: no information (not measured)
Incomplete outcome data (attrition bias)<br>: Hospital length of stay	Low risk	Judgement: all participants were included in the analysis
Incomplete outcome data (attrition bias)<br>: Postoperative quality of life	Unclear risk	Judgement: There is information but, the number of patients that used for this analysis was not shown
Selective reporting (reporting bias)	Unclear risk	Judgement: study protocol was not identified
Other source of bias	Low risk	Judgement: not detected

P. Liu 2013 [33]

Study characteristics	
Methods	**Study design:** prospective randomized double blind controlled trial<br>**Setting/country:** Multi-center (2)/China<br>**Date when study was conducted:** from April 2007 to December 2011
Participants	**Inclusion criteria**<br>• Age between 25 and 75 y<br>• Histologically confirmed colorectal cancer<br>• No distant metastasis<br>**Exclusion criteria**<br>• Age >75 y,<br>• Pregnancy<br>• Known lactose intolerance<br>• Clinically significant immunodeficiency<br>• Additional gastrointestinal disorders (eg, Crohn disease or ulcerative colitis)<br>• Use of antibiotics during the 10 d before surgery<br>• Evidence of infection<br>• Use of other pre-or probiotic or excessive fiber intake within 2 week of surgery<br>• History of an emergency operation<br>• Bowel preparation for colonoscopy within 6 d before surgery<br>• History of a proctectomy with low rectal anastomosis or surgery for a

	polypoid lesion<br>• Previous laparoscopic surgery<br>• Receipt of preoperative neoadjuvant chemotherapy or radiotherapy<br>• Distant metastasis.<br><br>**Total number of participants randomly assigned:** 161<br><br>**Probiotics**<br>• Number of all participant randomly assigned: 81<br>**Control**<br>• Number of all participant randomly assigned: 80
Interventions	**Intervention:** Probiotics<br>**Comparator:** Placebo<br>**Follow-up:** 1 month
Outcomes	**Outcomes**<br>• Mesenteric lymph node culture for bacterial translocation<br>• Intestinal permeability assay via lactulose/mannitol test<br>• Serum zonulin concentration<br>• Postoperative infectious complications (septicemia, central line infection, pneumonia, urinary tract infection, and diarrhea)<br>• Cumulative duration of antibiotic therapy<br>• Duration of postoperative pyrexia > 38.5<br>• Total hospital charge
Funding sources	None
Declarations of interest	None
Notes	**Protocol:** ChiCTR-TRC-00000423<br>**Language of publication:** English

Risk of bias		
Bias	Authors' judgement	Support for judgement
Random sequence generation (selection bias)	Low risk	Quote: "using a computer generated random allocation schedule"
Allocation concealment (selection bias)	Unclear risk	Quote: "treatment assignment was only known to a nurse who was not directly involved in the trial"
Blinding of participants and personnel (performance bias)<br>: All outcomes	Low risk	Quote: "double blinded, The appearance of the 2 types of capsules and the smell and taste of the study substances were the same"
Blinding of outcome assessment (detection bias)<br>: Subjective outcomes	Low risk	Quote: "double blinded"
Blinding of outcome assessment (detection bias)<br>: Objective outcomes	Low risk	Judgement: objective outcomes were unlikely affected by lack of blinding.
Incomplete outcome data (attrition bias)<br>: Perioperative mortality	Low risk	Judgement: all participants were included in the analysis
Incomplete outcome data (attrition bias)	Low risk	Judgement: all participants were included in the analysis

: Postoperative infectious complication		
Incomplete outcome data (attrition bias)<br>: Probiotics related adverse events	Unclear risk	Judgement: no information (not measured)
Incomplete outcome data (attrition bias)<br>: Postoperative overall complication	Low risk	Judgement: all participants were included in the analysis
Incomplete outcome data (attrition bias)<br>: Hospital length of stay	Unclear risk	Judgement: No information (not measured)
Incomplete outcome data (attrition bias)<br>: Postoperative quality of life	Unclear risk	Judgement: No information (not measured)
Selective reporting (reporting bias)	Unclear risk	Judgement: study protocol was not identified
Other source of bias	High risk	Judgement: the results in the full text article may be the results from a different study protocol performed by same author

Q. Zhang 2012 [34]

Study characteristics	
Methods	**Study design:** prospective randomized control study<br>**Setting/country:** single center/China<br>**Date when study was conducted:** from August 2006 to June 2007
Participants	**Inclusion criteria**<br>• Patients diagnosed with pathologically documented colorectal adenocarcinoma and intended to undergo elective radical CRC resection with laparotomy<br>• Patients aged aged 45 to 90 years<br>• No obvious contraindications to surgery<br>**Exclusion criteria**<br>• Neoadjuvant CRT<br>• If the radical resection failed or the CRC was complicated with malignant ascites<br>• Moderate to severe cardiac, pulmonary, hepatic or renal dysfunction<br>• Preexisting autoimmune disease<br>• Infectious disease within the 30 days preceding the enrollment<br>• If they had participated in any other clinical trial within the last 6 months<br><br>**Total number of participants randomly assigned:** 60<br><br>**Probiotics**<br>• Number of all participant randomly assigned: 30

	**Control**<br>• Number of all participant randomly assigned: 30
Interventions	**Intervention:** Probiotics<br>**Comparator:** Placebo<br>**Follow-up:** 1 month
Outcomes	**Outcomes**<br>• Changes in intestinal flora profile (E.coli, B. longum. ratio)<br>• Peripheral blood assay (IgG, M, A, IL-6, CRP, D-lactic acid<br>• Postoperative infectious complications (bacteremia. Septicemia, pneumonia, intra-abdominal abscess, SSI, perineal infection, anastomotic leak, fistula)<br>• Hospital length of stay
Funding sources	Shanghai Municipal Department of Health (2006045) and the Science and Technology Department of Zhejiang Province (2008C33039)"
Declarations of interest	None
Notes	**Protocol:** Not identified<br>**Language of publication:** English

Risk of bias

Bias	Authors' judgement	Support for judgement
Random sequence generation (selection bias)	Unclear risk	Judgement: not described
Allocation concealment (selection bias)	Unclear risk	Judgement: not described
Blinding of participants and personnel (performance bias)<br>: All outcomes	Low risk	Quote: "identically sealed with aluminum foil and administered in a double-blind manner"
Blinding of outcome assessment (detection bias)<br>: Subjective outcomes	Low risk	Quote: "identically sealed with aluminum foil and administered in a double-blind manner"
Blinding of outcome assessment (detection bias)<br>: Objective outcomes	Low risk	Judgement: objective outcomes were unlikely affected by lack of blinding.
Incomplete outcome data (attrition bias)<br>: Perioperative mortality	Low risk	Judgement: all participants were included in the analysis
Incomplete outcome data (attrition bias)<br>: Postoperative infectious complication	Low risk	Judgement: all participants were included in the analysis
Incomplete outcome data (attrition bias)<br>: Probiotics related adverse events	Unclear risk	Judgement: no information (not measured)
Incomplete outcome data (attrition bias)<br>: Postoperative overall complication	Low risk	Judgement: all participants were included in the analysis

Incomplete outcome data (attrition bias)<br>: Hospital length of stay	Low risk	Judgement: all participants were included in the analysis
Incomplete outcome data (attrition bias)<br>: Postoperative quality of life	Unclear risk	Judgement: No information (not measured)
Selective reporting (reporting bias)	Unclear risk	Judgement: study protocol was not identified
Other source of bias	Low risk	Judgement: not detected

CRC, colorectal cancer; CRT, chemoradiation therapy; E.coli, Escherichia coli; B.longum, Bifidobacterium longum; Ig, immumoglobulin; CRP, C-reactive protein; SSI, surgical site infection

R. Horvat 2010 [37]

Study characteristics	
Methods	**Study design:** prospective double-blind randomized placebo-controlled trial<br>**Setting/country:** single center/Slovenia<br>**Date when study was conducted:** Not reported
Participants	**Inclusion criteria**<br>• histologically confirmed diagnosis of adenocarcinoma of the colon<br>**Exclusion criteria**<br>• Age < 18<br>• Multiple malignancy of the colon<br>• Any clinical, laboratory or imaging signs of advanced malignant disease (invasion of adjacent organs, distant metastases)<br>• Signs of gastrointestinal obstruction<br>• History of inflammatory bowel disease<br>• Patients classified as ASA (American Society of Anesthesiologists) group > 3<br>• Patients who were not willing to participate in a study<br><br>**Total number of participants randomly assigned:** 60<br><br>**Synbiotic**<br>• Number of all participant randomly assigned: 20<br>**Prebiotic**<br>• Number of all participant randomly assigned: 28<br>**Control**<br>• Number of all participant randomly assigned: 20
Interventions	**Intervention:** Synbiotic<br>**Comparator:** standard care (no placebo)<br>**Follow-up:** 1 month
Outcomes	**Outcomes**<br>• Inflammatory marker (CRP, IL-6, leukocytes, fibrinogen and differential counts such as lymphocyte/granulocyte ratio)

	• Postoperative complications<br>• Hospital length of stay
Funding sources	Not reported
Declarations of interest	None
Notes	**Protocol:** Not identified<br>**Language of publication:** English

Risk of bias

Bias	Authors' judgement	Support for judgement
Random sequence generation (selection bias)	Low risk	Quote: "Randomization was achieved by random number sequence"
Allocation concealment (selection bias)	Unclear risk	Quote: "Sealed envelopes"<br>Judgement: There was no mention about 'opaque'
Blinding of participants and personnel (performance bias)<br>: All outcomes	High risk	Judgement: The control group did not receive a placebo.
Blinding of outcome assessment (detection bias)<br>: Subjective outcomes	Unclear risk	Judgement: not described
Blinding of outcome assessment (detection bias)<br>: Objective outcomes	Low risk	Judgement: objective outcomes were unlikely affected by lack of blinding.
Incomplete outcome data (attrition bias)<br>: Perioperative mortality	Unclear risk	Judgement: no information (not measured)
Incomplete outcome data (attrition bias)<br>: Postoperative infectious complication	Low risk	Judgement: all participants were included in the analysis
Incomplete outcome data (attrition bias)<br>: Probiotics related adverse events	Unclear risk	Judgement: no information (not measured)
Incomplete outcome data (attrition bias)<br>: Postoperative overall complication	Low risk	Judgement: all participants were included in the analysis
Incomplete outcome data (attrition bias)<br>: Hospital length of stay	Low risk	Judgement: all participants were included in the analysis
Incomplete outcome data (attrition bias)<br>: Postoperative quality of life	Unclear risk	Judgement: No information (not measured)
Selective reporting (reporting bias)	Unclear risk	Judgement: study protocol was not identified

Other source of bias	Unclear risk	Judgement: There is no statistical analysis of baseline characteristics

ASA, American Society of Anesthesiologists; CRP, C-reactive protein; IL-6, interleukin-6

S. Xia Yang 2010 [36]

Study characteristics	
Methods	**Study design:** prospective randomized controlled trial<br>**Setting/country:** single center/China<br>**Date when study was conducted:** from April 2008 to October 2008
Participants	**Inclusion criteria**<br>• Patients who diagnosed with CRC and scheduled to receive resection<br>**Exclusion criteria**<br>• History of IBD<br>• Severe heart, liver and kidney disorder<br>• Obstruction, perforation, bleeding<br>• Metastasis, unresectable disease status<br><br>**Total number of participants randomly assigned:** 60<br><br>**Probiotics**<br>• Number of all participant randomly assigned: 30<br>**Control**<br>• Number of all participant randomly assigned: 30
Interventions	**Intervention:** Probiotics<br>**Comparator:** standard care (no placebo)<br>**Follow-up:** 1 month
Outcomes	**Outcomes**<br>• Colonic mucosal change<br>• Postoperative complications<br>• Anastomotic leakage<br>• SIRS incidence<br>• Serologic marker (CRP)<br>• Duration of postoperative fever
Funding sources	Not reported
Declarations of interest	None
Notes	**Protocol:** Not identified<br>**Language of publication:** Chinese

Risk of bias		
Bias	Authors' judgement	Support for judgement
Random sequence generation	Low risk	Quote: "randomization principles according

(selection bias)		to the coin method"
Allocation concealment (selection bias)	Unclear risk	Judgement: not described
Blinding of participants and personnel (performance bias) : All outcomes	High risk	Judgement: The control group did not receive a placebo.
Blinding of outcome assessment (detection bias) : Subjective outcomes	Unclear risk	Judgement: not described
Blinding of outcome assessment (detection bias) : Objective outcomes	Low risk	Judgement: objective outcomes were unlikely affected by lack of blinding.
Incomplete outcome data (attrition bias) : Perioperative mortality	Unclear risk	Judgement: no information (not measured)
Incomplete outcome data (attrition bias) : Postoperative infectious complication	Unclear risk	Judgement: no information (not measured)
Incomplete outcome data (attrition bias) : Probiotics related adverse events	Unclear risk	Judgement: no information (not measured)
Incomplete outcome data (attrition bias) : Postoperative overall complication	Unclear risk	Judgement: no information (not measured)
Incomplete outcome data (attrition bias) : Hospital length of stay	Unclear risk	Judgement: no information (not measured)
Incomplete outcome data (attrition bias) : Postoperative quality of life	Unclear risk	Judgement: no information (not measured)
Selective reporting (reporting bias)	Unclear risk	Judgement: study protocol was not identified
Other source of bias	Low risk	Judgement: not detected

CRC, colorectal cancer; IBD, inflammatory bowel disease; SIRS, systemic inflammatory response syndrome; CRP, C-reactive protein

T. Zhang 2010 [35]

Study characteristics	
Methods	**Study design:** prospective randomized controlled trial

	**Setting/country:** single center/China<br>**Date when study was conducted:** from August 2006 to June 2007
Participants	**Inclusion criteria**<br>• Patients who diagnosed with CRC and scheduled to receive resection<br>**Exclusion criteria**<br>• Intestinal obstruction<br>• Perforation before surgery<br>• Enteritis<br>• Preoperative infectious status<br><br>**Total number of participants randomly assigned:** 60<br><br>**Probiotics**<br>• Number of all participant randomly assigned: 30<br>**Control**<br>• Number of all participant randomly assigned: 30
Interventions	**Intervention:** Probiotics<br>**Comparator:** standard care (no placebo)<br>**Follow-up:** 1 month
Outcomes	**Outcomes**<br>• Analysis of intestinal flora<br>• Detection of immune function and inflammatory response (IgG, M, A, IL-6, CRP, total number of lymphocytes)<br>• Fecal sIgA<br>• Postoperative infectious complications (incisional infection, abdominal, perineal infection, pulmonary infection, anastomotic leakage, postoperative intestinal function recovery, gastrointestinal adverse reactions)<br>• Hospital length of stay
Funding sources	Not reported
Declarations of interest	None
Notes	**Protocol:** Not identified<br>**Language of publication:** Chinese

Risk of bias

Bias	Authors' judgement	Support for judgement
Random sequence generation (selection bias)	Low risk	Quote: "random number table method"
Allocation concealment (selection bias)	Unclear risk	Judgement: not described
Blinding of participants and personnel (performance bias)<br>: All outcomes	High risk	Judgement: The control group did not receive a placebo.
Blinding of outcome assessment (detection bias)<br>: Subjective outcomes	Unclear risk	Judgement: not described
Blinding of outcome assessment (detection bias)<br>: Objective outcomes	Low risk	Judgement: objective outcomes were unlikely affected by lack of blinding.
Incomplete outcome data	Unclear risk	Judgement: no information (not measured)

(attrition bias)<br>: Perioperative mortality		
Incomplete outcome data (attrition bias)<br>: Postoperative infectious complication	Low risk	Judgement: all participants were included in the analysis
Incomplete outcome data (attrition bias)<br>: Probiotics related adverse events	Low risk	Judgement: all participants were included in the analysis
Incomplete outcome data (attrition bias)<br>: Postoperative overall complication	Low risk	Judgement: all participants were included in the analysis
Incomplete outcome data (attrition bias)<br>: Hospital length of stay	Unclear risk	Judgement: There are comments about the outcome, but the exact result is not presented.
Incomplete outcome data (attrition bias)<br>: Postoperative quality of life	Unclear risk	Judgement: No information (not measured)
Selective reporting (reporting bias)	High risk	Judgement: Results of anastomotic leakage were not reported.
Other source of bias	Low risk	Judgement: not detected

CRC, colorectal cancer; IgG, immunoglobulin G; IL-6, interleukin-6; CRP, C-reactive protein; fecal sIgA, fecal secretory IgA

U. Gianotti 2010 [38]

Study characteristics	
Methods	**Study design:** randomized, double-blind clinical trial<br>**Setting/country:** multi center (two) / Italy<br>**Date when study was conducted:** from October 2006 to October 2007
Participants	**Inclusion criteria**<br>• Patients with histological documentation of cancer of the colon and rectum<br>• Age between 18 and 80 years<br>• Candidate for elective colorectal resection<br>**Exclusion criteria**<br>• Denied written informed consent<br>• No collection of a stool sample 4 d before the operation<br>• Unresectable tumor, neoplastic ascites<br>• Clinically relevant pulmonary<br>• Cardiovascular, hepatic and kidney dysfunction or failure<br>• Ongoing total parenteral nutrition

	• Immunological disorders<br>• Ongoing or recent infections (within last 30 d)<br>• Pregnancy<br>• Participation to another clinical trial.<br><br>**Total number of participants randomly assigned:** 32<br><br>**Probiotics**<br>• Number of all participant randomly assigned: 21 (high dose: 10, low dose:11)<br>**Control**<br>• Number of all participant randomly assigned: 10
Interventions	**Intervention:** Probiotics<br>**Comparator:** Placebo<br>**Follow-up:** Not mentioned
Outcomes	**Outcomes**<br>• Primary Efficacy Parameters: morphological and microbiological evaluation of the colonic microflora, gastrointestinal function<br>• Immune and inflammatory response, bacterial translocation
Funding sources	Not reported
Declarations of interest	None
Notes	**Protocol:** NCT00936572<br>**Language of publication:** English

Risk of bias		
Bias	Authors' judgement	Support for judgement
Random sequence generation (selection bias)	Low risk	Quote: "central randomization by computer"
Allocation concealment (selection bias)	Low risk	Quote: "Both probiotic and placebo preparations were in foil sealed sachets that were stored in identical numbered containers. The study products and the placebo were both white powders, identical in weight, smell, and taste. Thus, the identity of the specific product was blind to participants, support staff and investigators for the entire duration of the study period."
Blinding of participants and personnel (performance bias)<br>: All outcomes	Low risk	Quote: "double-blind, Data entering, from CRFs into a computer database, was blinded. The blind codes were broken after all the collected data were analyzed."
Blinding of outcome assessment (detection bias)<br>: Subjective outcomes	Low risk	Quote: "double-blind, Data entering, from CRFs into a computer database, was blinded. The blind codes were broken after all the collected data were analyzed."
Blinding of outcome assessment (detection bias)<br>: Objective outcomes	Low risk	Judgement: objective outcomes were unlikely affected by lack of blinding.
Incomplete outcome data	Unclear risk	Judgement: no information (not measured)

(attrition bias)<br>: Perioperative mortality		
Incomplete outcome data (attrition bias)<br>: Postoperative infectious complication	Unclear risk	Judgement: No information (not measured)
Incomplete outcome data (attrition bias)<br>: Probiotics related adverse events	Unclear risk	Judgement: No information (not measured)
Incomplete outcome data (attrition bias)<br>: Postoperative overall complication	Unclear risk	Judgement: No information (not measured)
Incomplete outcome data (attrition bias)<br>: Hospital length of stay	Unclear risk	Judgement: No information (not measured)
Incomplete outcome data (attrition bias)<br>: Postoperative quality of life	Unclear risk	Judgement: No information (not measured)
Selective reporting (reporting bias)	Low risk	Judgement: Not detected
Other source of bias	Low risk	Judgement: not detected

CRF, case report form
