# Supplementary material for: Perioperative Probiotics Application for Preventing Postoperative Complications in Patients with Colorectal Cancer: A Systematic Review and Meta-Analysis
Source: Medicina (Kaunas). 2022 Nov 14;58(11):1644. doi: 10.3390/medicina58111644 (PMC9699544; doi:10.3390/medicina58111644)
Supplement: Supplementary file 1 [file medicina-58-01644-s001.zip › supplementary_file_5_exc_study.pdf]

Table S5. Characteristics of excluded studies.

Study	Reason for exclusion
Carlini 2022 [40]	wrong study design (not randomized controlled trial, retrospective study)
Zaharuddin 2019 [19]	wrong intervention (taking probiotics 4 weeks after colorectal cancer surgery)
Tarvirdizade 2019 [43]	wrong study population (no information about disease entity)
Xu 2019 [47]	wrong intervention (probiotics + glucose solution)
Golkhalkhali 2018 [45]	wrong study population (participants who received chemotherapy)
Consoli 2016 [41]	wrong study population (participants with benign colorectal disease)
Magnell 2012 [42]	wrong study population (participants with benign colorectal disease)
Oliveira 2012 [39]	wrong study design (not randomized controlled trial, prospective longitudinal study)
Worthley 2009 [44]	wrong study population (participants with healthy volunteer)
Liu 2015 [46]	wrong study population (participants who underwent primary colorectal cancer and liver metastasis)
